# A Review of the Metal Additive Manufacturing Processes

**DOI:** 10.3390/ma16247514

**Published:** 2023-12-05

**Authors:** Mohaddeseh Tebianian, Sara Aghaie, Nazanin Sadat Razavi Jafari, Seyed Reza Elmi Hosseini, António B. Pereira, Fábio A. O. Fernandes, Mojtaba Farbakhti, Chao Chen, Yuanming Huo

**Affiliations:** 1 School of Metallurgy and Materials Engineering, Iran University of Science and Technology (IUST), Narmak, Tehran 13114-16846, Iran; 2 TEMA: Centre for Mechanical Technology and Automation, Department of Mechanical Engineering, University of Aveiro, Campus de Santiago, 3810-193 Aveiro, Portugal; 3 State Key Laboratory of Powder Metallurgy, Central South University, Changsha 410083, China; 4 School of Mechanical and Automotive Engineering, Shanghai University of Engineering Science, Shanghai 201620, China

**Keywords:** additive manufacturing, metal 3D printing, deposition

## Abstract

Metal additive manufacturing (AM) is a layer-by-layer process that makes the direct manufacturing of various industrial parts possible. This method facilitates the design and fabrication of complex industrial, advanced, and fine parts that are used in different industry sectors, such as aerospace, medicine, turbines, and jewelry, where the utilization of other fabrication techniques is difficult or impossible. This method is advantageous in terms of dimensional accuracy and fabrication speed. However, the parts fabricated by this method may suffer from faults such as anisotropy, micro-porosity, and defective joints. Metals like titanium, aluminum, stainless steels, superalloys, etc., have been used—in the form of powder or wire—as feed materials in the additive manufacturing of various parts. The main criterion that distinguishes different additive manufacturing processes from each other is the deposition method. With regard to this criterion, AM processes can be divided into four classes: local melting, sintering, sheet forming, and electrochemical methods. Parameters affecting the properties of the additive-manufactured part and the defects associated with an AM process determine the method by which a certain part should be manufactured. This study is a survey of different additive manufacturing processes, their mechanisms, capabilities, shortcomings, and the general properties of the parts manufactured by them.

## 1. Introduction

The additive manufacturing (AM) process (or 3D printing), as a new and strategic process, has attracted much attention in the industry in the last years and, as a result, has been growing drastically. Typically, AM is a layered manufacturing process [1,2,3] that includes forming and processing of material. It is worth mentioning that this technique is fully computer-controlled [4].

According to the ASTM 52900 [5], the AM process is defined as “a method of the joining of materials to fabricate parts from 3D-model data, made layer upon layer. This process is opposed to formative manufacturing and subtractive manufacturing methodologies”.

**Figure 1:**
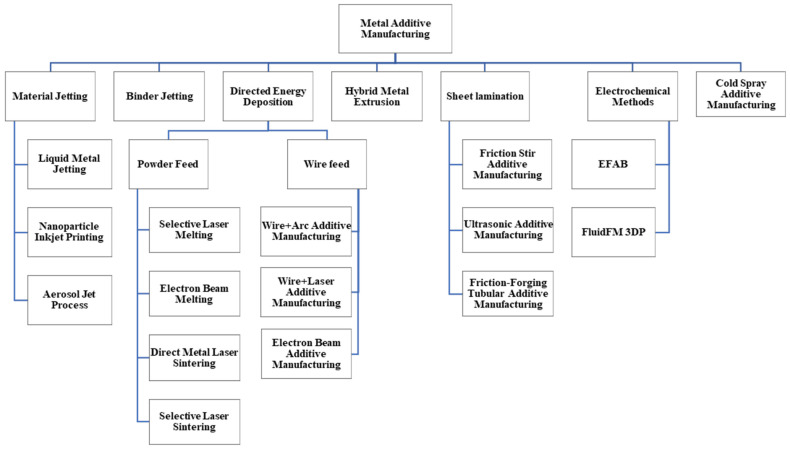
A classification of the principal current metal AM techniques [13,14].

Additive manufacturing is a process based on discrete stacking, which is controlled via software and a material control system. Generally, 3D-manufactured parts are made by stacking multiple layers. Hence, this process is so flexible that high-speed manufacturing can be achieved while having minimum material waste. One of the main advantages of the AM process is the production of complex-structured parts that find application in aerospace, medical industries, etc., because the traditional manufacturing processes for these specific parts are extremely limited. This extensive attention toward this process has motivated many researchers to develop new advanced metal additive manufacturing techniques [6,7,8,9]. A classification of the metal AM processes is presented in Figure 1.

These processes have been classified with regard to various criteria like the form of utilized material (e.g., wire, powder, etc.), types of heating sources (e.g., laser, arc, electron beam, etc.), build volume, etc. According to the above-mentioned classifications, metallic additive manufacturing processes can be divided into multiple methods like SLA, SLS, SLM, WAAM, etc. [2,10,11,12]. The main purpose of this study is to present the classification and introduction of metal AM processes based on the heating source, materials, and deposition methods.

## 2. Material Jetting

Material Jetting (MJ) is a class of AM techniques. In this process, the feedstock droplets of the materials are deposited selectively [1].

### 2.1. Liquid Metal Jetting

Liquid Metal Jetting (LMJ) is a solid freeform production technique for making polymeric, ceramic, and metallic parts as well as electronic interconnects. LMJ is a process like inkjet printing in which molten droplets are printed. By controlling the solidification speeds and the composition of the alloy, LMJ can create parts with outstanding properties. This method leads to the production of dense parts with a finer grain structure that enhances the mechanical properties. In the case of aluminum, the use of this method increases the yield strength by 30% compared to the other jetting methods. Liquid metal jet printing (LMJP) is an emerging production process that addresses several challenges in the solid freeform fabrication (SFF) method. The method is based on the technology analogous to inkjet printing. Unlike the spray deposition and spray forming techniques that spray the materials in an uncontrolled route, the LMJP controls and dispenses every single molten droplet of the material to a determined location by computer-aided design (CAD) data. Among the parameters affecting the properties of the final part fabricated by this method, the droplet exit speed, size, shape, and solidification speed can be mentioned. The possible applications of the LMJP involve the ability to quickly fabricate 3D electronic circuitry and mechanical parts [13,15,16].

**Figure 2:**
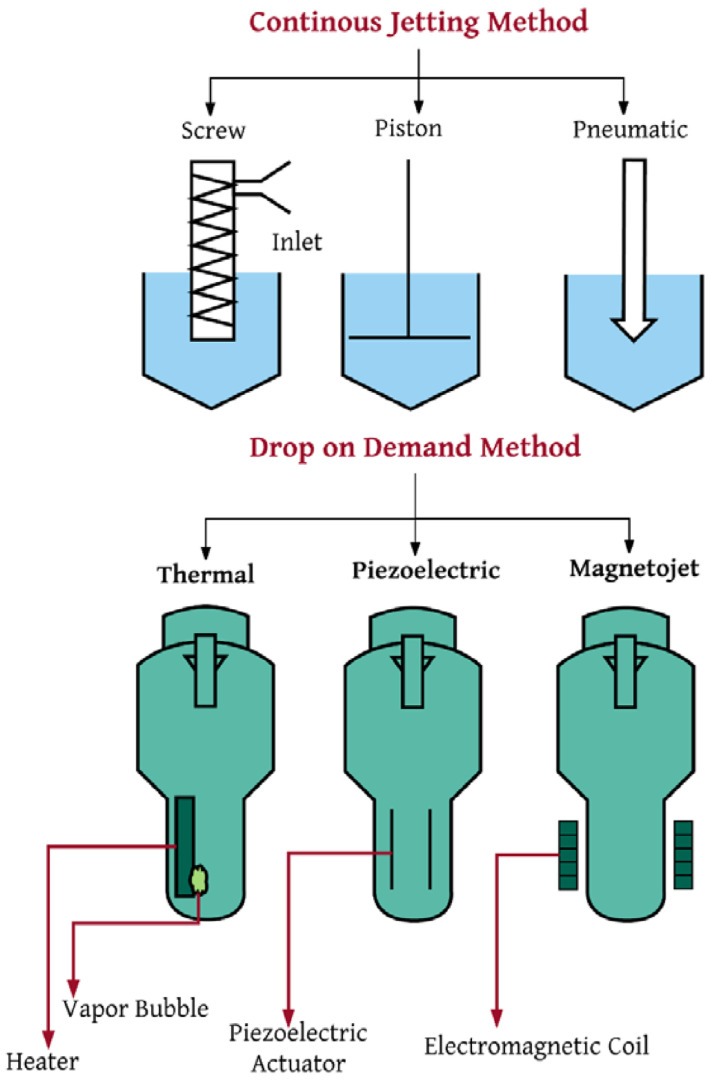
Continuous and drop-on-demand methods of jetting 3D printing methods [17].

There are two jetting techniques: drop-on-demand and continuous jetting. The schematics of these processes are shown in Figure 2. Continuous jetting is applied when the material is continuously jetted. A thin-liquid jet is examined to be discontinuous if the jet breaks up, and then the droplet formation takes place at the orifice or nozzle of the jet. Table 1 depicts the differences between drop-on-demand and continuous methods and their applications. The utilization of this method has increased the printing speed and reduced the costs compared to other additive manufacturing methods. Also, the volume of the formed melt is not a limiting factor, and it is possible to recycle the melt in this method. Oxidation is one of the problems in this technique, which can be overcome by using the drop method [16,17,18].

### 2.2. Nanoparticle Inkjet Printing

**Figure 3:**
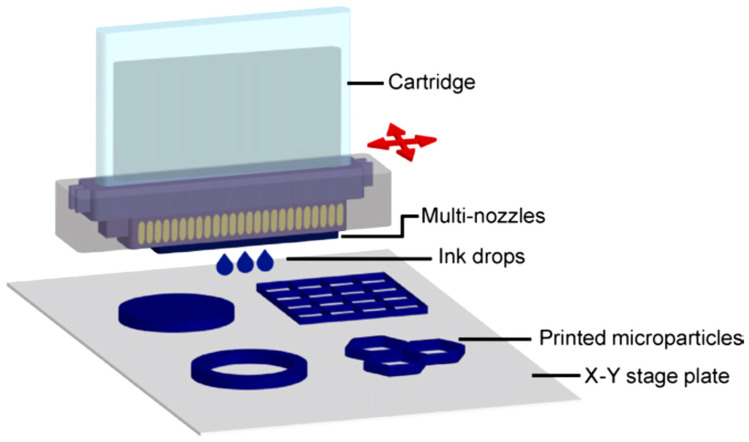
Schematic of the piezoelectric inkjet system [22].

The inkjet printing method is a deposition method applied for the liquid phase materials. These materials or inks involve a solute-dissolved material or a dispersed material in a solvent. This technique includes the ejection of a specific amount of the ink in the chamber from a nozzle and quasi-adiabatic reduction of the volume of the chamber through piezoelectric action. The chambers filled by the liquid are contracted in response to the utilization of the external voltage. These sudden reductions set up a shockwave in liquid and make a liquid drop eject from nozzles. A schematic of this technique is depicted in Figure 3 [19]. The deposition process of the inkjet printing may also be performed continuously (as opposed to drop-wise deposition of the material) [8,20].

Nanoparticles possess several exceptional properties that are different from the bulk material. The thermodynamic size lowers the nanoparticles’ melting point compared with the bulk material. This property of the nanoparticles is much more useful for flexible electronics. In order to obtain highly conducting printed tracks, metallic nanoparticles like gold, silver, and copper are utilized. Silver nanoparticles become an appropriate material for ink formulation compared to gold, especially for obtaining low cost, low resistivity, and low oxidation rates [7,21].

### 2.3. Aerosol Jet Printing

**Figure 4:**
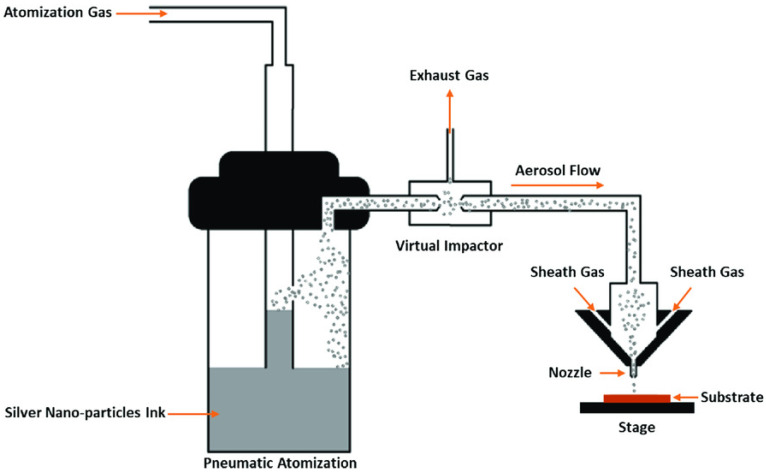
Schematic of the system of AJP [23].

Aerosol Jet Printing (AJP) is the spraying of ink containing small size droplets that are dispersed in the liquid (Figure 4). The technique involves two components: an atomizer and a deposition head. An atomizer is an ultrasonic or pneumatic device that produces a dense vapor of droplets. A carrier gas like nitrogen moves [10,23] across the atomizer in order to transfer the dense vapor into the deposition head section. The resulting material flow leaves the head from a nozzle onto the substrate. This process is suited for 3D utilizations because the deposition head could be mounted to a 5-axis positioning stage to follow the substrate contour at 1 to 5 mm of fixed distance. Furthermore, acquiring fine characteristics is achievable because the aerosol involves a large density of micro-droplets, which are focused on fabricating lines with thickness values of about 10 μm [11,24,25,26].

## 3. Powder Bed Fusion

**Figure 5:**
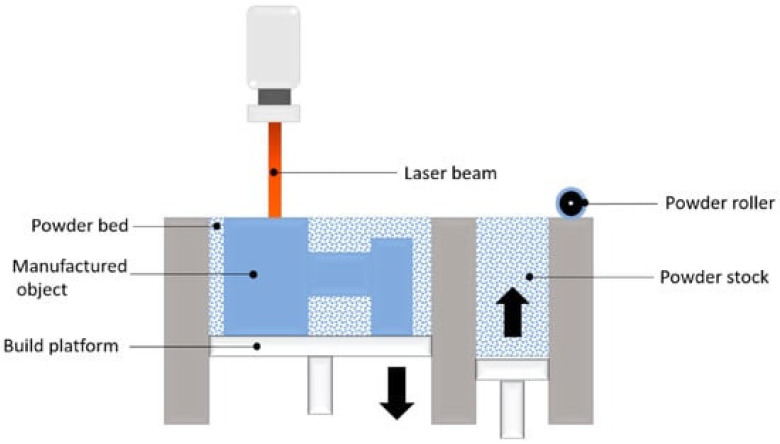
Schematic of the PBF technique [28].

Powder Bed Fusion (PBF) methods are based on coating a metal powder layer with a thickness of 20 to 200 microns on a platform and then scanning the powder bed with a source of heat that melts and then solidifies the powder along the beam path. The layer-by-layer laser scanning is managed by the CAD program of the part to be manufactured. Figure 5 depicts the schematic steps of this process [14,27,28,29].

### 3.1. Selective Laser Melting

**Figure 6:**
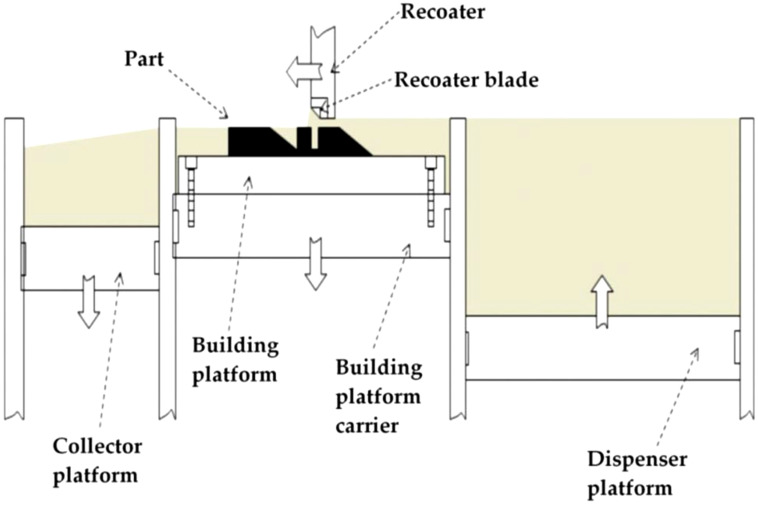
A schematic of the SLM machine [30].

Selective laser melting (SLM) is one of the PBF techniques that are the much widely utilized in the AM industries due to their high production speed, covering a wide range of materials, and the possibility of powder recycling. This method is commonly used for the production of chromium and titanium alloys and stainless steels, especially in medical applications. A layer of metallic powders is utilized to coat the building platform on the previously deposited layer or the substrate by a blade and then melted by a laser beam. The laser beam melts the powders as programmed by the CAD data. Afterward, the building platform moves to the downside, and then a new layer of the powder is subjected to the laser. The technique is repeated until the height of the part is completed. In the SLM process, powders with particle sizes of 20 to 50 µm are used, and each layer thickness value varies from 15 µm to 150 µm. Many parameters must be considered carefully to produce a defect-free sample. Some of the main parameters of the powder are the shape, size, fluidity, laser scan speed, laser power, hatch overlaps, hatch distance, and hatch style, which have an important influence on the mechanical properties of the produced parts. The technique is performed inside a chamber. The chamber is filled with Ar or N_2_ as inert gases. The used gas depends on the metal powder reactivity. Moreover, the chamber is subjected to overpressure situations. The existence of the inert gas in the chamber reduces the oxidization during this process. The substrate plate is preheated between 200 °C to 500 °C to minimize the solidification rate in the produced parts [30,31,32,33,34]. A schematic of the SLM technique is depicted in Figure 6.

The SLM process imparts superior mechanical properties compared to the SLS technology as a result of the complete melting of the powder. The main disadvantage of SLM compared to SLS is the surface tension of the melt, which makes the process more difficult to control. This technology is known for producing high-precision complex parts with high resolution in the range of 250 to 700 µm. Among the disadvantages of SLM, time-consuming post-processing, dependence on the powder morphology, high electricity consumption, and creation of structural defects such as anisotropy, gas entrapment, lack of fusion, and the micro-porosity (less than 2%) can be mentioned (Table 2) [35,36].

Zhang et al. [39] investigated the effect of selective laser melting energy density on the stainless steel 316 L. According to their studies, among the factors that determine the energy density, the laser scanning speed and laser power have the greatest influence on the grain size of porous scaffolds. As the energy density increases, the distance between primary dendrites increases and the microhardness decreases. Moreover, low residual stress was reported at low energy density values.

### 3.2. Electron Beam Melting

**Figure 7:**
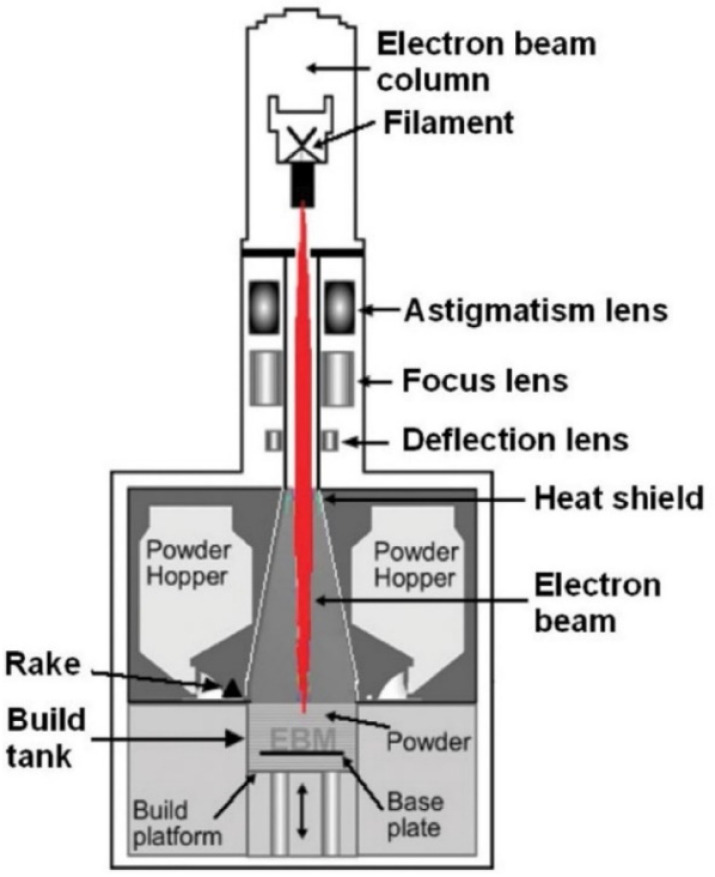
A schematic of the EBM process [30].

Electron Beam Melting (EBM) technology was developed by researchers from Chalmers University of Technology in Sweden in 2003 [40]. The EBM process (Figure 7) applies the electron beam energy to melt the metallic powders. Each layer is provided by the following steps: spreading the metallic powders, preheating and then sintering the powder with a defocused beam that obtains mechanical stability as well as electrical conductivity to the metallic powder layer, melting the powders by using a focused beam, and lowering the building platform by the thickness of one layer which may vary from 50 µm to 200 µm.

The process occurs in a vacuum with a pressure of roughly 10^−5^ mbar and at high temperatures. The materials produced with EBM have microstructural features that are better than those of wrought and cast materials because this method produces stress-relieved materials with microstructures free from martensitic features. The helium gas with a partial pressure of 2 × 10^−3^ mbar is introduced to the EBM chamber during the melting process to protect the chemical specification of the produced material. Therefore, EBM is suited for the production of materials with a high affinity to reacting with O_2_ like Ti alloys [29,30,41].

The EBW process has a higher speed than SLM and provides the possibility of making complex parts. Some of the advantages of this method are the possibility of contamination protection, less residual stress and shrinkage, and freedom in design. The EBM is currently the preferred process for metal fabrication; however, it is limited by the electrical conductivity of the material and the need to perform the process in a vacuum. The disadvantages of this method include higher fatigue cracking of the produced samples, electrostatic charge of the powder, and rougher surface compared to SLM samples. The comparison of the surface roughness, porosity, and the thickness of layers of some processes are shown in Table 2 [38,42].

Murr et al. [43] investigated the mechanical properties of IN 625 superalloy made by the EBW method under different conditions. Based on the results, the yield strength in the normal EBW condition (as fabricated sample) was reported to be 9% lower than the annealed condition, while the ductility did not change. Furthermore, in HIPing conditions, the ductility is increased by 57%, while the yield strength is decreased by 20%.

Layer-by-layer deposition techniques lead to the creation of structures that intensify the possibilities of fatigue crack initiation and have a significant impact on fatigue life. The fatigue life of Ti-6Al-4V alloy parts fabricated by EBM and LBM was investigated. The results of the study illustrated that the LBMed Ti-6Al-4V alloy has a longer fatigue life than the EBMed part. The difference in the fatigue life behavior may be attributed to the surface features (higher roughness values). High surface roughness sites act as the fatigue crack initiation sites in EBM materials. In EBW, the surface roughness is higher than the LBW (Table 2) and as a result, its fatigue life is lower than the EBW [44].

### 3.3. Direct Metal Laser Sintering

**Figure 8:**
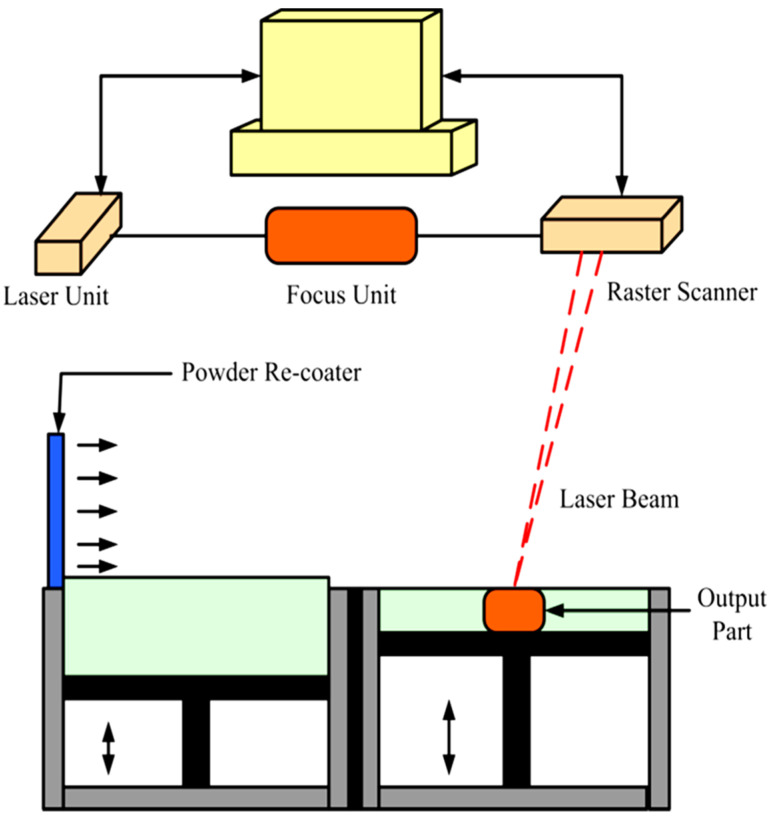
A schematic diagram of the DMLS technique [46].

With the development of high-energy-density lasers in the mid-1990s [45], the development of the Direct Metal Laser Sintering (DLMS) process was initiated. DMLS is an advanced laser-based additive manufacturing process that utilizes 3D-design data to create a part by a layer-by-layer consolidation route. DMLS initiates using the application of a thin layer of metallic powder on the platform. As depicted in Figure 8, a laser beam with a high-power liquid phase sinters each layer of the powder. Afterward, the build platform moves and is lowered, and then the re-coater blade spreads the powders on the platform after each scanning. Because of its flexibility in shapes and feedstock, this process provides notable potential for producing complex products that cannot be manufactured by other techniques. DMLS is a much more effective process among different types of AM processes that can manufacture any complex shape. Some advantages of the DMLS process are less wastage of powder materials, less surface roughness than EBW (Table 2), and the material variety that can be utilized in this technique such as Ti6Al4V, AlSi10Mg, IN 718, aluminum alloys, etc. [38,45,46,47].

### 3.4. Selective Laser Sintering

**Figure 9:**
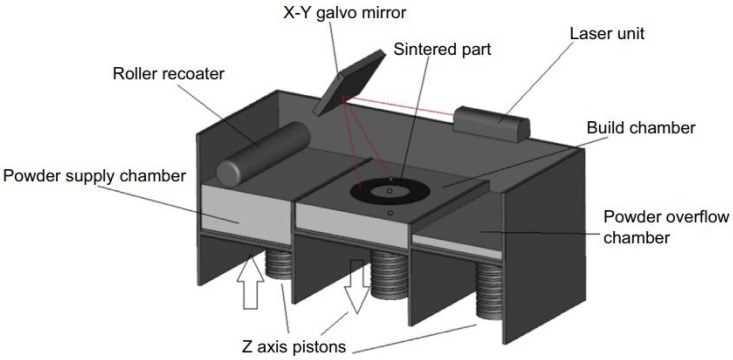
A schematic of the SLS process [51].

The Selective Laser Sintering (SLS) technique is a method that fabricates layers with predesigned geometry by sintering the powders with the laser beam (Nd-YAG or CO_2_). This method was developed in 1989 for the additive manufacturing of polymers and has been recently used widely for metals [45]. The technique steps are as follows: (1) the substrate is moved down to a depth equal to the layer thickness; (2) a layer of powder is rolled out on the substrate; and (3) the laser scans deposited a layer of powder to sinter powders at the determined area. Stages 1, 2, and 3 are repeated until the designed sample is completed. A schematic of SLS is depicted in Figure 9 [48,49,50].

## 4. Binder Jetting

Binder jetting (BJ) is an AM technology capable of handling alloys such as Cu-, Al-, Ni-, Fe-, and Co-based alloys, bronze, brass, and gray iron, as well as ceramics such as glasses, sand, and graphite. However, this process could use any material that is available in powder form and allows color printing. This process applies two materials: one is the ceramic/metal-based material of which the part is to be built, and the other is the binder material that glues the ceramic/metal powders within and between the layers [31]. The binder is normally liquid, while the ceramic/metal powder is in the form of a solid. In this process, the ceramic/metal powder is firstly spread on a substrate, and then a layer of the binder is deposited on the ceramic/metal layer. This is performed by the CAD model. The BJ process includes some post-processing like infiltration, sintering, depowering, curing, and finishing. The post-processing usually takes a longer time compared with the actual printing, especially in the sintering part. One of the important advantages of this process is that the products can be manufactured without the support structures [52,53]. The advantages and disadvantages of this method are summarized in Table 3.

**Figure 10:**
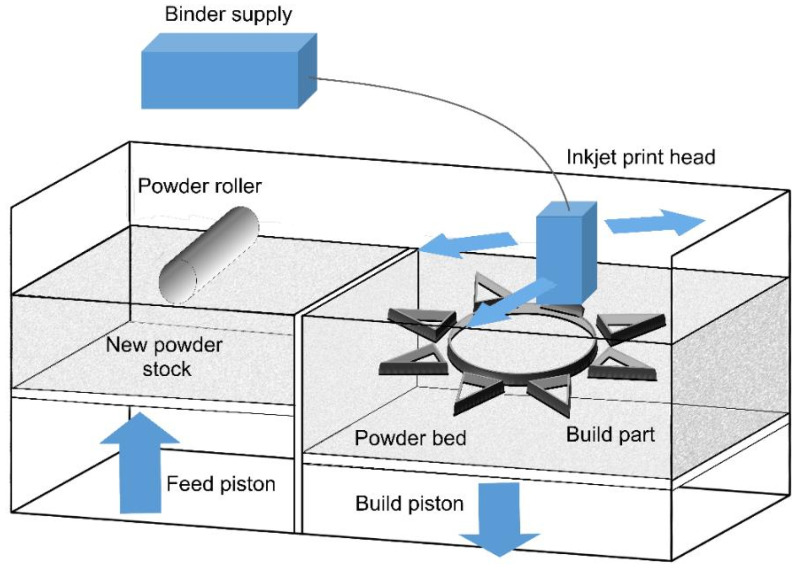
The schematic of the binder jetting process [57].

As BJ utilizes binders as the adhesive materials, the material characteristics are not always suitable for automobile and aerospace applications because the binder could lead to porosity formation in conventional sintering techniques. The speed of this technique (12–24 mm/h) is quicker than that of the EBM/SLM techniques and could be accelerated by the increase in the number of the print head holes, which deposit the binder and the material. This also allows the two-material approach in which different binder–powder combinations lead to various mechanical properties by changing the binder-to-powder ratio [31,37,56]. The process schematic is illustrated in Figure 10.

In this route, a powder layer is first spread by a counter-rotating roller. Then, the inkjet printing head sprays the liquid agent on the bed in order to fabricate the 2D pattern for each layer. Some powder/binder systems can utilize heaters in order to control the curing and moisture. However, the heat is not a fundamental requirement in the process. After making each layer (minimum layer thickness of 0.09 m) [37], the build platform is moved down to provide room for the next layer. As-printed parts are normally brittle and usually are subjected to post-processing to improve their mechanical properties [54,58]. The maximum build volume of this method is 4000 × 2000 × 1000 mm^3^ [37].

Nowadays, a large percentage of industrial BJ manufacturing is associated with metallic materials. Many of these processes focus on the powder metallurgy of alloys like stainless steels. Many industrial applications need high-density alloys. Although high densities have been obtained in different materials, this is still a challenge to decrease defects and provide accuracy in the geometry of the sample. The maximum level of density obtained for the powders of steel after the sintering is about 92–95%. The Hot Isostatic Pressing (HIP) process is a technique to densify powder-based products to over 99% density [54,57].

## 5. Cold Spray Additive Manufacturing

**Figure 11:**
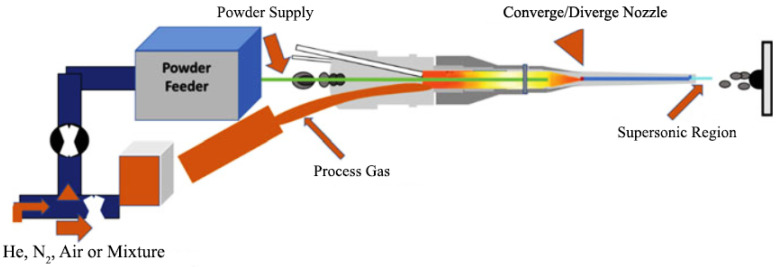
A gun schematic of a cold spray process [59].

Cold Spray Additive Manufacturing (CSAM) is a method in which solid-state particles are delivered to the substrate through a carrier gas with a supersonic speed to deposit a layer on the substrate. Figure 11 depicts a schematic of this method. High speed is a main requirement for particle deposition and achieving dense coating. Parameters such as nozzle geometry, particle characteristics, and gas conditions affect the velocity. This process is suitable for thermally sensitive coatings such as nano-crystalline, amorphous materials, and oxygen-sensitive materials like titanium, aluminum, and magnesium composites. The application of this process is the surface upgrading of metals to increase the corrosion resistance and wear and electrical/thermal conductivity. Because this method is performed at low temperatures, it is suitable for coating metals such as magnesium. The process could be designed in either a manual or an automated operation mode. Gases with aerodynamic properties such as N_2_, He, combinations of N_2_ and He, and dry air (21% O_2_ + 79% N_2_) are used in this process [59,60,61].

The main components of the process include the following: powder feeder and spray gun; gas source; the pre-nozzle entry of the gas heater to compensate for the cooling due to quick nozzle expansion; a supersonic nozzle; a spray chamber with a motion mechanism; and the monitoring and controlling the gas temperature and pressure of the spraying.

**Figure 12:**
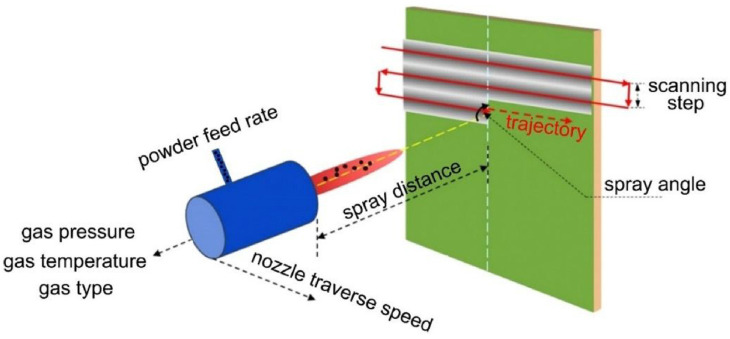
The components schematic of the cold spray technique [63].

Figure 12 represents cold spray components. Compressed gases like He, O_2_, and N_2_ pass through a system that consists of a powder feeder and a gas heater at pressure values in the range of 1.4–3.4 MPa, which is maintained at about 1.7 MPa. The gas is heated electrically at 100–600 °C and then travels across a diverging/converging nozzle till it reaches the supersonic speed. The powder is subjected to the gas stream before the converging zone of the nozzle. The extending gas accelerates the technique. A decrease in the temperature happens In the middle of the supersonic expansion of the nozzle. Consequently, the temperature of the gas stream is almost below the particles’ melting point, which develops a solid-state coating from particles without any oxidation [60,62].

**Figure 13:**
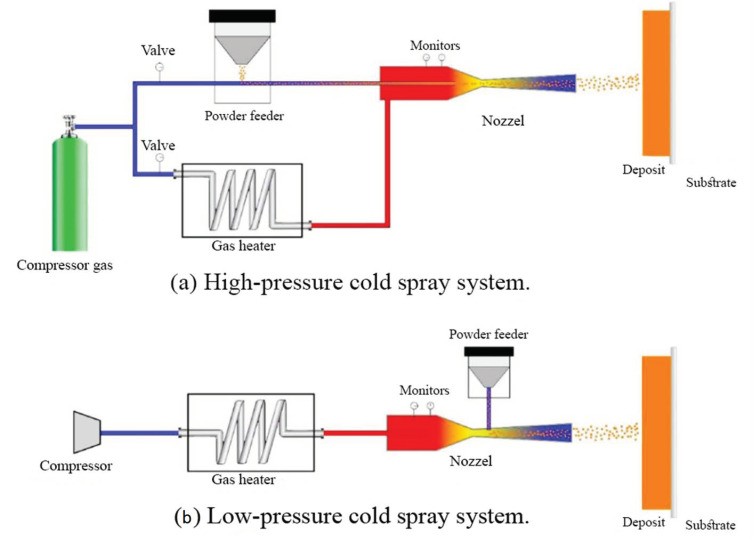
Schematic of (**a**) high- and (**b**) low-pressure cold spray processes [63].

The cold spray technique could be divided into two classes based on the propulsive gas pressure: low-pressure cold spray (less than 1 MPa) and high-pressure cold spray (more than 1 MPa). Figure 13a depicts a schematic of a high-pressure cold spray process. Compressed gas is separated into two streams before entering the spray system. One stream, which is named propulsive gas, proceeds across a gas heater and is warmed to a high temperature. The second stream, which is named carrier gas, goes across the powder feeder, where it is loaded with the feedstock. Then, the aforementioned gas streams are mixed before entering the nozzle and expanding to produce a supersonic gas and also a powder stream. Figure 13b represents the schematic of a low-pressure cold spray process [61,63].

The CSAM process has similarities with some manufacturing processes such as extrusion, laser AM, and friction stir welding. This process imparts anisotropic properties due to its layering and particle distribution. The extent of his anisotropy is partially dependent on the inherent properties of the matter. For this reason, more malleable metals such as aluminum and copper are used. This process is generally used for coating and has not been investigated on a large scale. This method has a lower production rate and poor mechanical properties compared to friction stir welding. In the CSAM process, high hardness has been observed due to the impact of the projectile motion of the powder. Meanwhile, other properties such as plasticity, mechanical strength, electrical conductivity, and abrasion properties are weakened, which can be attributed to the micro-porosity and intergranular boundaries formation [37,64,65].

## 6. Hybrid Metal Extrusion and Bonding Additive Manufacturing

Hybrid Metal Extrusion and Bonding Additive Manufacturing (HYB-AM) is a new solid-state technique that uses metal wire to deposit the metal in a stringer-like manner in order to make layers and produce a net-shaped structure. After the deposition, the produced part is finished by the machining to create the desired net shape [64].

**Figure 14:**
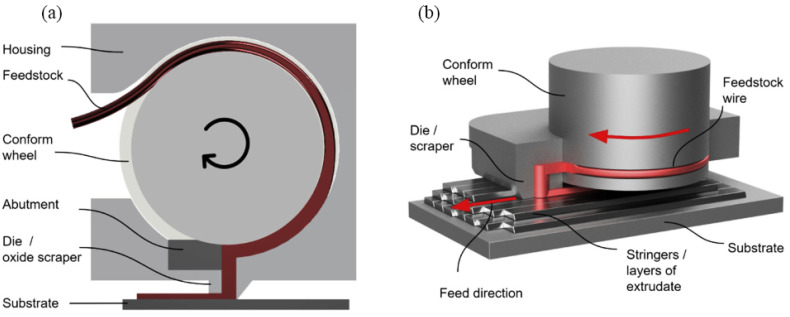
A schematic of (**a**) an HYB-AM extruder and (**b**) an extruder and the deposited structure [65].

**Figure 15:**
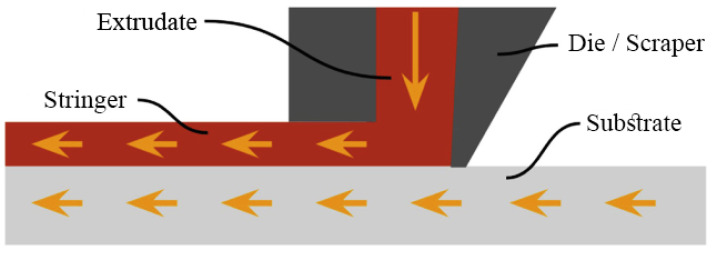
A schematic of the bonding achieved between the substrate and the extrudate at the pressure exceeding the materials’ flow stress in the HYB-AM process [65].

The flow of the material in this method is based on continuous rotary extrusion, which is also recognized as Conform extrusion. The extrusion sequence involves two steps. In the first step, the feedstock is deformed in an extruder. This step causes the oxides present on the feedstock surface to become dispersed into the extrudate. In the second step, the extruder supplies the pressure to acquire bonding at the interface between the underlying structure and the extrudate. According to Figure 14a, the extrusion pressure is produced by the frictional force between the tapered groove and the feedstock wire in the rotating wheel. The feedstock is compressed into the groove and is also driven forward by the wheel rotation. Then, the feedstock wire is blocked by the abutment and axial compression is induced. This causes the material to be yielded and fill the cross-section. This enhances the contact surface and also the friction and leads to a build-up in further pressure and causes the material to flow out of the die. Figure 14b represents the HYB-AM deposition process. The extruder adds the material as it shifts in the deposition direction and places stringers side-by-side to create a layer. Then, it allows new layers to be built on the top of the material. The die is constantly scrapping the underlying layer top during deposition, and then the adjacent stringer side wall removes the surface oxide. Before the deposition, a metal strip is fixed on the heated bed to behave as a substrate on the first layer of the deposited material. In the HYB-AM process, the merging metal streams must be considered as the mating streams of substrate and extrudate, as shown in Figure 15 [65,66].

## 7. Sheet Lamination

The sheet lamination processes used in metal additive manufacturing are divided into three categories: ultrasonic additive manufacturing (UAM), friction stir additive manufacturing (FSAM), and friction-forging tubular additive manufacturing (FFTAM) techniques [67].

### 7.1. Ultrasonic Additive Manufacturing

The UAM technique utilizes metallic sheets, which are bound together by using ultrasonic welding. Different alloys used in this process include copper alloys, aluminum alloys, titanium alloys, and stainless steels. The process happens at a low temperature. One advantage of this process is that the technique can bond various materials. Meanwhile, it requires little energy. The alloy is not melted. The layers are bonded together using a combination of ultrasonic oscillation and pressure [68].

**Figure 16:**
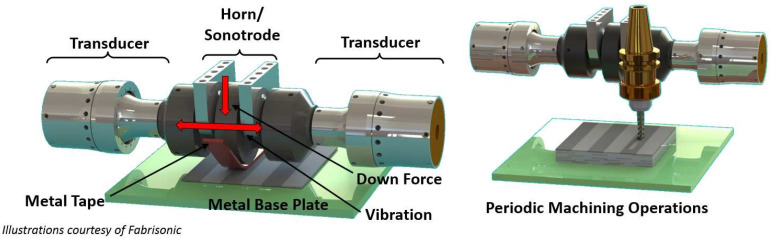
Schematic of an ultrasonic AM machine [70].

This process which is also recognized as Ultrasonic Consolidation (UC) is a hybrid method that combines ultrasonic metal seam welding and computer numerical control (CNC) milling. In this method, the part is built up on the base plate, which is rigidly bolted onto the heated plate. The temperature of this plate ranges from room temperature to approximately 200 °C. Parts are manufactured in a bottom-to-top manner. Furthermore, each layer is built up of metal tape-like foils laid side-by-side and then cut utilizing CNC. As depicted schematically in Figure 16, in this process, a rotating sonotrode advance along a length of thin metal foil (usually 100–150 μm thick) while applying a normal force to the new layer, keeping it in close contact with a base plate/previous layer. This ultrasonic oscillatory force is applied transversely to the motion direction at a constant frequency of 20 kHz and a user-set amplitude. After the deposition of a foil, another foil is deposited right next to the previous one. This process is repeated till the completion of a layer. All these deposited layers experience the same procedure. Each level of UAM consists of four layers of deposited metallic foils. After one level of deposition, the CNC head cuts the deposited layers to their contour. It should be noted that the geometry of the part dictates the contouring procedure. The consecutive addition–subtraction of layers is continued until the final geometry of the part is achieved. Furthermore, each layer is deposited as an assemblage of foils laid side-by-side rather than a single continuous sheet, unlike the other sheet lamination processes [67,69].

### 7.2. Friction Stir Additive Manufacturing

**Figure 17:**
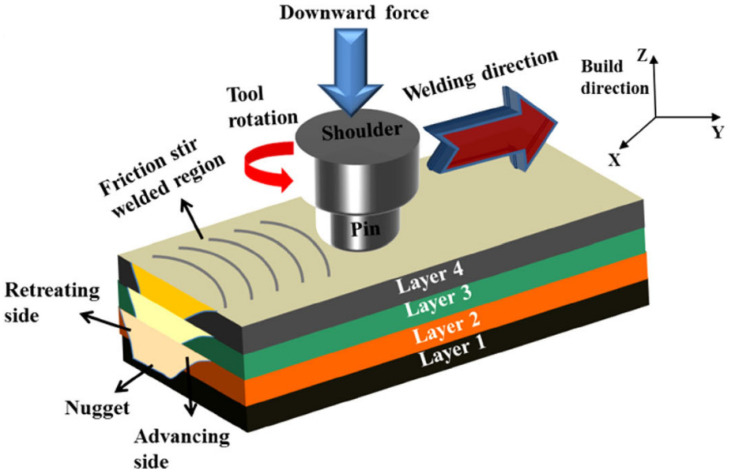
Schematic arrangement of FSAM [74].

FSAM falls into the category of solid-state additive manufacturing processes that could be considered a combination of layer-by-layer FSW and AM processes. Owing to its exceptional ability for microstructural engineering and grain refinement, it can be useful in tuning microstructures to fit the customer’s requirements. It is reported that high-strength alloys could be fabricated by this process. In FSAM, the fundamental layer-by-layer AM is employed. Hence, a stack of overlapping sheets/plates is penetrated by a consumable tool, and the FSLW process is conducted along the defined direction. Figure 17 illustrates a schematic diagram for the FSAM process. Friction and plastic deformation in a workpiece create enough heat for the joining of the layers. The joint creation is made possible as a result of the material transfer, heat generation, and material consolidation from the front to the tool’s rear parts. In addition to process parameters, the tool geometry during FSAM also partially defines the macroscopic and microscopic aspects of the manufactured parts [71,72,73].

**Figure 18:**
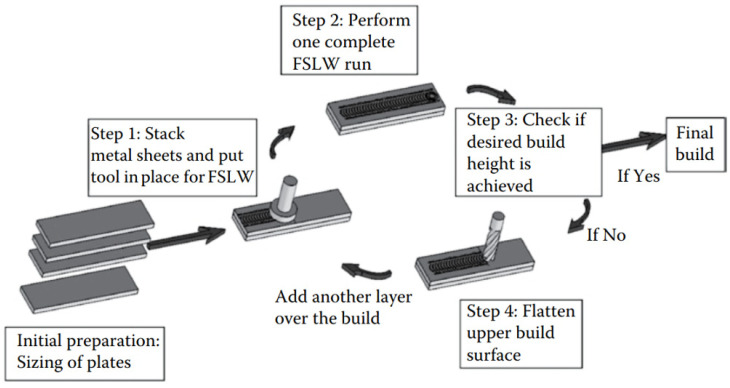
Steps utilized in FSAM [75].

The sequential steps carried out in the FSAM process are illustrated in Figure 18 and can be described as follows:

1. The plates/sheets that are additively manufactured are prepared with regard to surface properties. These plates are manufactured in the desired dimensions and degreased with the acetone.
2. Stacking metal sheets: In this step, two plates should be overlapped, one over the other, and oriented as desired.
3. Performing a complete FSLW run: After the stacking of the two sheets/plates, the FSLW is performed. After the first run, provided the required build height is made, the process will be finalized. Otherwise, the process will proceed to step 4.
4. Flattening of the upper surface: If the required build height is not made, the deposition of new layers over the build is needed. Therefore, the upper surfaces of the previously fabricated layers are flattened in order to remove the flash that occurred during FSLW. After surface preparation, a new sheet/plate is placed over the top layer. Then, steps 2–4 are repeated until the desired height of the build is provided [75].

### 7.3. Friction Forging Tubular Additive Manufacturing

**Figure 19:**
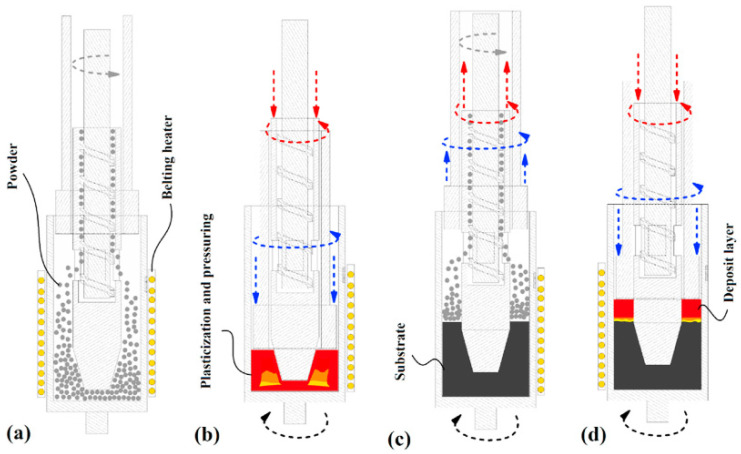
FFTAM method steps: (**a**) the introduction of the powder, (**b**) powder pressing by forging and rotational friction stirring, (**c**) the addition of the next layer of the powder, and (**d**) the new layer pressing under friction forging (gray arrows: rotation direction of the inert part, red: rotation direction and movement of the inert tools; blue: rotation direction and movement of the outer tools) [71].

A solid-state, three-dimensional production method similar to the FSAM technique could be suggested in which the materials are plasticized; therefore, tubular structures can be deposited layer by layer. This newly invented processing route is referred to as friction-forging tubular additive manufacturing (FFTAM). The schematic of Figure 19a–d shows different steps for the deposition of layer-upon-layer of metallic materials in the FFTAM method. As depicted in the schematic diagram of Figure 19a, first of all, the powder mixture in the form of chips are inserted from the storage chamber into the free space of the central shoulder cavity by the screwing action of a rotating screw mandrel. After inserting the material, the principal hollow cylindrical punch with a groovy surface forges the powder and consolidates it via rotational movement, as represented in Figure 19b. Through conducting such thermomechanical treatment, the powder mixture/chips are plasticized drastically and then form a solid layer. In order to apply the maximum shear strain at an elevated temperature, the rotational directions for the main outer hollow sheath/shoulder and cylindrical punch have to be in the opposite direction with respect to each other. Chip consolidation can be enhanced at high temperatures and shear strains through a deformation-assisted diffusion mechanism at hydrostatic pressure. It should be pointed out that in the constant heat input, excessive ejection of the powder chips/mixture can result in the unsuccessful deposition of layers caused by incomplete solidification of feeding material. Meanwhile, lower amounts of the powder chips/mixture for each layer could lead to overheating along the thickness and cause hot cracking between them. Consequently, the feeding powder volume is a critical parameter. The optimization of rotational speed for the visceral punch, main outer shoulder, and the number of feeding chips per deposition layer is obtained via several trials and errors. According to Figure 19a, it is possible to manufacture a short tube through several passes of the layer-upon-layer deposition technique [71].

## 8. Direct Energy Deposition

Further, 3D cladding and 3D welding are the main techniques in the direct energy deposition (DED) additive manufacturing technique used for the production of low-carbon steels, stainless steels, aluminum, titanium, and nickel alloys. In 3D cladding, a laser or plasma beam melts the metal powder ejected from the feeding nozzle in order to form a layer, and 3D welding, also called shaped metal deposition (SMD), is a wire-based technique in which a small-diameter wire is fed and then melted, binding to the previous layers via welding. The summary of the features and parameters of this method is presented in Table 4.

**Figure 20:**
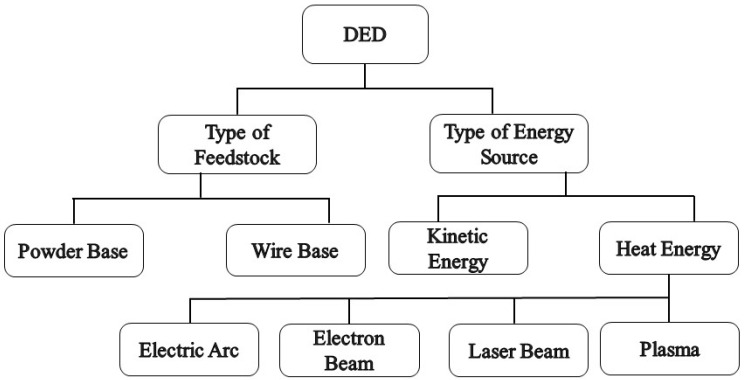
Classification of directed energy deposition systems [77].

There are different types of direct energy deposition systems including wire feed and powder feed-based DED (based on the type of feedstock), kinetic energy-based DED (based on the source type of the energy), and melt-based DED. The melting DEDs can be categorized based on the plasma, laser, electric arc, and electron beam. The flowchart in Figure 20 shows the different DED classes. Some important points can be given as follows: the DED process takes place during several stages, which include placing a substrate on the worktable. In the case of using the laser method, the chamber of the device is filled with inert gas, and in the case of using the electron beam process, a vacuum is used to reduce the oxygen level in the chamber. At the beginning of the method, the electron or laser beam creates a molten pool on the surface. The material transfer is conducted using a nozzle (laser as powder and beam as wire). The nozzle and the beam move along the path determined by the CAD data. The successive layers are melted and frozen on each other until the process is completed [37,58,76,77,78].

### 8.1. Powder Feed Systems

**Figure 21:**
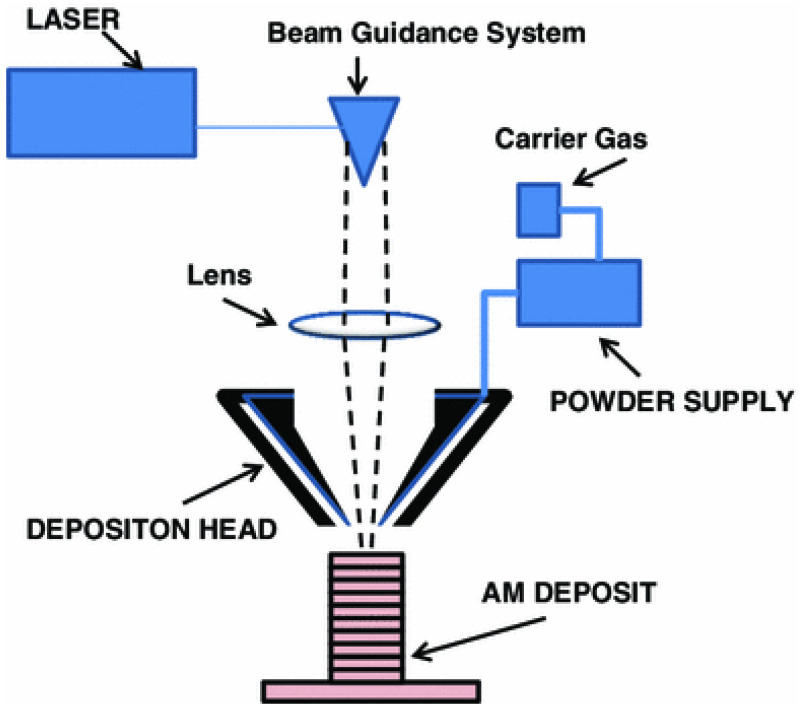
Schematic of the powder feed system [38].

Figure 21 depicts a schematic of the laser-based metal deposition of the DMD technique. Utilizing the powder feeders, numerous organizations have tried to develop powdered DED machines, which are also recognized as laser engineered net shaping (LENS), direct metal deposition (DMD), and laser consolidation (LC). Even though the general method seems to have no variation, subtle nuances have been distinguished, including the laser powder, the laser type, the laser spot size, the powered delivery method, the feedback control scheme, the inert gas delivery method, and/or a type of motion control. The final parts seem to acquire a dense structure during the building process because all these processes are comprised of the deposition, the melting, and the solidification of the powder consuming a traveling melt pool. A wide range of lasers have been utilized in the laser-based processes including CO_2_ laser, Nd: YAG laser, diode lasers, and fiber laser. The LENS technique was originated by the Sandia National Laboratories in 1997. Afterwards, it was licensed to Optomec (USA), as the DMD technique was developed by the POM group and the University of Michigan jointly. In the meantime, LENS and DMD technologies offer the ability to deposit different materials in a single build and the ability to add metal to existing parts [38,58,79,80].

Accufusion laser consolidation is quite analogous to the LENS process, where a powder is deposited into a molten metal bath using a laser that provides the required energy for the deposition. Similar to the LENS method, the laser consolidation is carried out in a tightly sealed chamber. This process produces better as-built surface finishes than the LENS systems; however, it suffers from lower deposition rates. Table 5 shows a list of equipment suppliers and the specifications of their equipment [81].

### 8.2. Wire Feed System

The deposit volume always equals the fed wire volume in the wire feeding, and there is near-unity feedstock capture efficiency (if the “splatter” from the melt pool is neglected). As simple geometries, “blocky” geometries are without many thick/thin transitions, and the most effective coatings are mainly fabricated by wires [41,82].

#### 8.2.1. Wire Arc Additive Manufacturing

**Figure 22:**
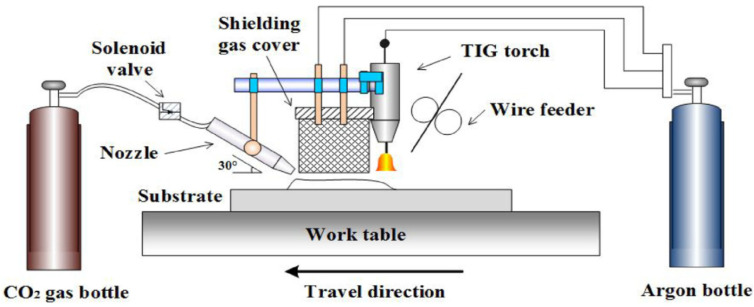
Basic WAAM process [10].

Wire arc additive manufacturing (WAAM) is one of the modern methods among the additive manufacturing processes of metals and is also recognized as shaped metal deposition (SMD) or rapid plasma deposition (RPD). The schematic of this method is demonstrated in Figure 22. The main components of this system include a wire feeding, welding torch, power source, and computer system, which are used to control the arc, deposition rate, and wire feeding [10].

In the WAAM process, three methods are conventional to provide the heat input, which includes the metal inert gas (MIG), tungsten inert gas (TIG), and plasma arc welding (PAW). Among these methods, using the MIG process is easier and more convenient than the other two methods because of the connection of the wire with the welding torch. In PAW and TIG, an external system is required to transfer the wire [82,83].

For WAAM-fabricated Ti-6Al-4V parts, the yield strength and tensile strength in the Z direction (normal to the deposition plane) were lower than in the X-Y plane. Moreover, the fatigue cracks initiated in the pores near the surface and the porosity were attributed to the absorption of N, O, and H gases in the molten deposits [84,85].

#### 8.2.2. Wire Laser Additive Manufacturing

**Figure 23:**
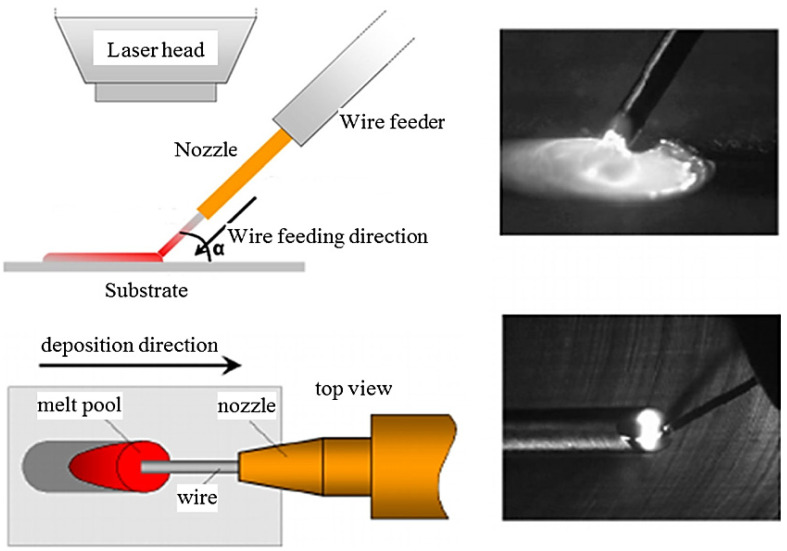
A schematic of the wire laser additive manufacturing and top-slide view images of the real process [82].

Wire laser additive manufacturing (WLAM) is capable of producing full-density metal by utilizing metal wires as the additive metallic material and the laser source energy. This system is composed of multiple parts, an automatic wire-feeding system, a laser, and a computer-controlled worktable. As depicted in Figure 23, the laser creates the melting pool and molten wire metal simultaneously. Then, by feeding the molten wire to the melt pool, a metallurgical bonding with the substrate can be formed. By movement of the laser processing head and the wire feeder on the substrate, the bead-shaped solid is formed. The performance in the WLAM process is determined by many terms like surface finish, the geometry and quality of the deposit, the final microstructure of the deposited layer, and the resulting mechanical properties [82,86,87].

#### 8.2.3. Electron Beam Additive Manufacturing

**Figure 24:**
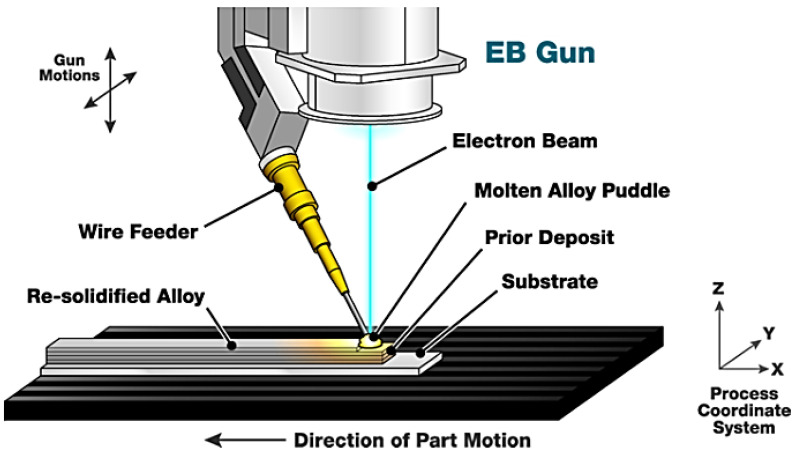
Basic electron beam additive manufacturing process [90].

Electron beam additive manufacturing (EBAM) can create and sustain a molten pool using a focused electron beam in a high-vacuum chamber. It is worth mentioning that the feeding material in this process is mainly wire metal. CNC sequencing offers a singular or integral combination of movement involving a part, electron beam, or wire feeder, which facilitates the forming of complicated structures. The principal advantage of EBAM compared to other DED technologies is the prevention of surface oxidation, which leads to higher purity of deposited layers. Another benefit of this process is superior and faster beam control through electromagnetic lenses, which eases the deposition rate increase in electrically conductive materials, even in highly reflective alloys like aluminum and copper, and improves the sensitivity index. The sensitivity index is defined as the ratio of the component volume and the size of the actual deposition (Figure 24) [82,88,89].

## 9. Electrochemical Methods

It is reported that, currently, there are two electrochemical metal AM techniques, electrochemical fabrication (EFAB) and Fluid FM 3DP.

### 9.1. Electrochemical Fabrication

**Figure 25:**
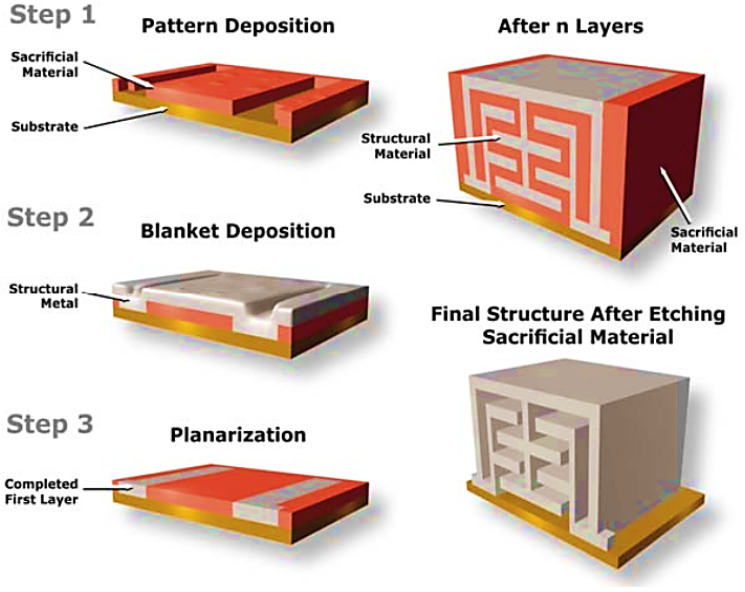
The EFAB method. A three-step method is used on each layer by using two materials. Eventually, one material is etched in order to release the structure [91].

Electrochemical fabrication (EFAB) is a recent solid freeform fabrication (SFF) process with high economic efficiency for fabricating prototypes or different parts on a massive scale. EFAB generates a whole layer simultaneously versus serially, like most SFF processes. EFAB can be utilized to form structures from electrodepositable metals. Based on the electrodeposition, the EFAB can deposit ultra-thin layers (2–10 µm or thinner) with minimized stairsteps, which leads to a fully dense metal structure with the possibility of being homogeneous and isotropic. The minimum deposited linewidth is about 25 µm, which can be further reduced. Electrochemical fabrication can be used to fabricate micromachines and micro-electro-mechanical systems (MEMS), offering high efficiency and considerable advantages over the current methods including true 3D geometry, compatible IC, process automation, and low capital investment. In order to build 3D micro-objects with the EFAB method, layer-by-layer electrochemical deposition and subtractive planarization are used. Each layer deposition involves three main steps: electroplating of sacrificial support material through a special method known as “instant masking”, electroplating of build material, and planarization. The steps for the manufacturing of the metal parts in the EFAB technique are illustrated in Figure 25. By placing the first layer as a sacrificial metal, all devices are fully separated from the substrate. It should be pointed out that this process is usually utilized for the fabrication of medical devices. Nowadays, the EFAB technique is compatible with three fully commercialized and specific materials like Ni–Co alloy, Valloy™-120, Edura™-180, palladium, and a rhodium formulation. These materials could be utilized for several applications because of their diverse functionality. It is worth noting that it is possible to use other materials in the EFAB technique depending on the final application requirements [91,92].

### 9.2. Fluidic Force Microscope

**Figure 26:**
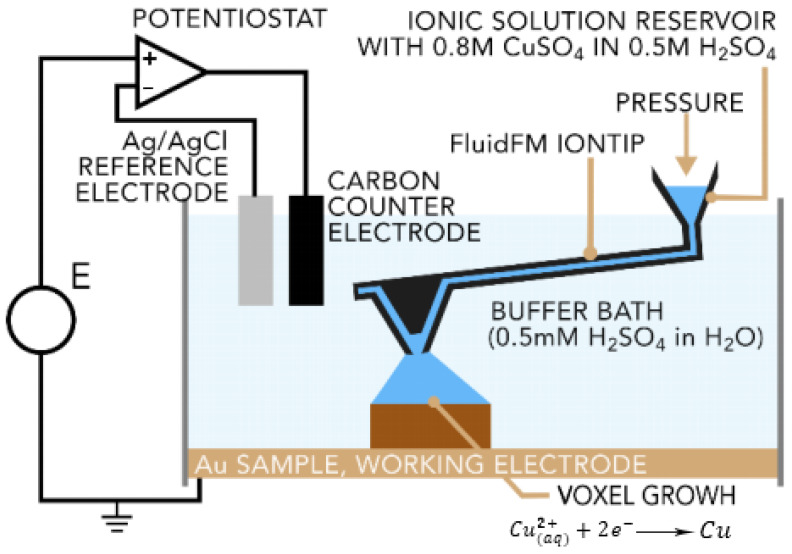
Fluid FM printing cell. An ion tip dispenses the electrolyte into a bath of a buffer. At the working electrode, Cu^2+^ ion is deposited as a solid copper [14].

Cynosure AG has recently developed an electrochemical technique known as the Fluidic Force Microscope (Fluid FM) process. In this process, electro-deposition, precise liquid ink, and scanning probe microscopy (SPM) dispense the coalescence. This technique makes use of atomic force microscopy (AFM) cantilevers with a microfluidic channel and a hollow tip. The electrolyte solution contained in a reservoir is pushed through the cantilever and out of the tip via the application of pressure by a microfluidics control system (Figure 26). The tip is a part of the printing head that can move in three dimensions inside the buffer bath. The metal cations are deposited to form the solid metal through the application of a proper potential to the built substrate. A real-time feedback system is employed for the detection of any tip deflection. As the formation of a voxel is completed, the voxel upper face interacts with the tip, exerting a force that leads to a few-nanometer cantilever deflection. When the deflection reaches a certain amount, the voxel is considered to be printed. Meanwhile, the tip moves to the next voxel [14,93].

## 10. Summary

Metal additive manufacturing processes are relatively new methods that allow the production of complex structures. In this review, AM processes were categorized based on their deposition methods, and then they were investigated from a broad perspective by focusing on the process steps, materials, and heat source types. In AM processes, feeding materials can exist in many forms such as powder, bulk wire, foil, etc. The feeding system, even for a single type of the feed material, may vary from one process to another. The computer control system plays a major role in the automating of AM processes, resulting in higher surface quality and geometrical accuracy. Some advanced technologies of the heat sources like the electron beam, the laser beam, and the laser–arc hybrid used in these methods make them an interesting spectrum of solutions for manufacturing challenges.

## Figures and Tables

**Table 1:** Differences between drop-on-demand (DOD) and continuous methods and their utilizations [16].

Parameters	Drop-on-Demand	Continuous
Jet Speed (droplet numbers per second)	Less than 10 kHz	10–100 kHz in a cylindrical configuration and 5 to 20 (kHz) in a pump configuration.
Drop Size Relative to Orifice Size (diameter to diameter)	Same, which is better for smaller drops producing	Droplets are 1.8 times bigger than the orifice diameter that is better for producing larger drops.
Material Usage	Less	Unwanted droplets should be guttered. Unused materials can be reused in various applications.
Generator (Force/Energy Required)	More	Less

**Table 2:** Comparison of commercialized SLM, EBM, and DLMS processes [37,38].

System	Process	Build Volume (mm)	Energy Source	Surface Roughness (µm)	Layer Thickness (µm)	Porosity
Concept laser	SLM	300 × 350 × 300	200 or 400 W	5–15	10–100	Low than 2%
Phenix system	SLM	245 × 245 × 360	200 W			
Arcam AB	EBM	200 × 200 × 350	7 kW electron beam	20	50–200	Low than 1%
EOS	DMLS	250 × 250 × 325	200–400 W Yb-fiber laser	5–16	20–100	2–5%

**Table 3:** Some disadvantages and advantages of the binder jetting technique [54,55].

Advantages	Disadvantages
Fast process	Low density
Wide range of materials	Its application limited to metals.
High build size	Need for post-processing

**Table 4:** Summary of the features and parameters of the DED technique [37,76].

Particle size (µm)	40–110
Beam spot (µm)	660–5000
Power range (W)	300–1000
Scanning speed (mm/s)	1–20
surface roughness (µm)	30.6–63.9
Advantage	Wide range of materials High rate of deposition and fabrication. Can be used to fabricate relatively bulky parts. High density Economical.
Disadvantage	Fabrication of complex geometries is challenging. High surface roughness. Need for post-processing The control of the process is the difficult task. High residual stress
Defect	Cracking Delamination residual stress Porosity

**Table 5:** Representative powder-based DED equipment suppliers and specifications [81].

System	Method	Building Volume (mm)	The Source of Energy
Optomec (LENS 750)	LENS	300 × 300 × 300	500 W, 1 kW or 2 kW IPG fiber laser
Optomec (LENS 850-R)	LENS	900 × 900 ×1500	1 or 2 kW IPG-fiber laser
POM DMD (66R)	DMD	3200° × 360° × 3670°	1–5 kW fiber-diode/disk-laser
Accufusion laser consolidation	LC	1000 × 1000 × 1000	Nd: YAG laser

## Data Availability

Not applicable.
